# Expression of concern “Selective mapping of brain COX-1 with [11C]PS13: Pharmacokinetic evidence from human PET imaging”

**DOI:** 10.1016/j.ibneur.2025.06.001

**Published:** 2025-06-03

Expression of concern for ‘Selective Mapping of Brain COX-1 with [11C]PS13: Pharmacokinetic Evidence from human PET Imaging’ by Orangi et al., IBRO Neurosci. Rep., 18 (2025), pp. 657–662, https://doi.org/10.1016/j.ibneur.2025.04.012.

The Editors are issuing this Expression of Concern to inform readers that significant textual overlap and paraphrasing have been identified between this article and 'Whole-Body PET Imaging in Humans Shows That ^11^C-PS13 Is Selective for Cyclooxygenase-1 and Can Measure the In Vivo Potency of Nonsteroidal Antiinflammatory Drugs' by Kim et al., J Nucl Med 2023; 64:159–164, https://doi.org/10.2967/jnumed.122.264061.

The authors were contacted for an explanation; however, the responses from the corresponding authors, Dr. Mahsa Asadi Anar and Dr. Niloofar Deravi, were deemed unsatisfactory. The Editors have requested all data used in Orangi et al. (2025), details on the methods of obtaining summary data from Kim et al. (2023) and the OpenNeuro dataset (ds004868), as well as a thorough description of the analytic methods employed, be submitted within thirty (30) days. This Expression of Concern will remain associated with the article until a conclusive outcome is reached.

